# Caregiver Quality of Life When Persons Living with Dementia Spend Time in Healthcare Settings

**DOI:** 10.1093/geroni/igaf122.2155

**Published:** 2025-12-31

**Authors:** Cassandra Dictus, Justine Seidenfeld, Wenhan Zhang, Kevin McKenna, Nathan Boucher, Megan Shepherd-Banigan, Susan Hastings, Courtney Van Houtven

**Affiliations:** Duke University, Durham, North Carolina, United States; Durham VA Healthcare System, Durham, North Carolina, United States; Duke University, Durham, North Carolina, United States; Duke University, Durham, North Carolina, United States; Duke University, Durham, North Carolina, United States; Duke University, Durham, North Carolina, United States; Durham VA Healthcare System, Durham, North Carolina, United States; Duke University, Durham, North Carolina, United States

## Abstract

Dementia significantly impacts the quality of life (QoL) of both persons living with dementia (PLWD) and their family caregivers. Efforts are underway to develop person-centered measures that reflect both PLWD and caregiver well-being. Caregivers play a crucial role in helping PLWD remain at home, which is associated with higher PLWD QoL. However, recent research suggests that measuring PLWD home time (days spent at home versus facility-based care) does not fully reflect caregiver QoL. Understanding how PLWD time in different health care settings affects caregiver QoL is essential for developing meaningful, caregiver-centered measures. We interviewed 46 family caregivers (mean age 68 years, 70% female, 96% spouses) of PLWD with experiences in emergency, inpatient, outpatient, post-acute, and assisted living settings. Using a qualitative descriptive approach with directed content analysis, we identified key factors influencing caregiver QoL. These include caregiver financial/social resources, transportation, view of PLWD’s well-being during the stay, time spent staying with PLWD in the setting, as well as setting-specific elements such as the quality of care, communication with staff, physical environment, and access to specialists. Caregivers’ experiences were complex with positive and negative QoL impacts such as relief from worry, new stressors, improved sleep, exhaustion from driving back and forth, respite from caregiving, and frustration with new care tasks, such as managing insurance or care coordination. Findings highlight the need for refined caregiver QoL measures beyond home time. Understanding caregiver perspectives is vital to developing person-centered metrics that inform interventions and policies aimed at supporting both PLWD and their caregivers.

